# A word of hope for ataxia trials in COVID-19 time and beyond

**DOI:** 10.1007/s00415-020-10231-9

**Published:** 2020-09-22

**Authors:** Roderick P. P. W. M. Maas, Jordache Ramjith, Thomas Klockgether, Kit C. B. Roes, Bart P. C. van de Warrenburg

**Affiliations:** 1 Department of Neurology, Donders Institute for Brain, Cognition, and Behaviour, Radboud University Medical Center, Reinier Postlaan 4, 6525 GC Nijmegen, The Netherlands; 2 Department of Health Evidence, Section Biostatistics, Radboud University Medical Center, Nijmegen, The Netherlands; 3 Department of Neurology, University of Bonn, Bonn, Germany; 4 German Center for Neurodegenerative Diseases (DZNE), Bonn, Germany

**Keywords:** Cerebellar ataxia, Randomized controlled trial, COVID-19, Scale for the assessment and rating of ataxia

## Abstract

The coronavirus disease 2019 (COVID-19) crisis confronted us, like many researchers worldwide, with an unforeseen challenge during the final stages of a randomized controlled trial involving ataxia patients. Institutional guidelines suddenly no longer allowed regular follow-up visits to take place, impeding the clinical evaluation of long-term outcomes. Here, we discuss the various scenarios that we considered in response to these imposed restrictions and share our experience of home video recording by dedicated, extensively instructed family members. Albeit somewhat unconventional at first glance, this last resort strategy enabled us to reliably assess the study’s primary endpoint at the predefined point in time and hopefully encourages researchers in other ongoing ataxia trials to continue their activities. Remote assessments of ataxia severity may serve as a reasonable substitute in interventional trials beyond the current exceptional situation generated by the COVID-19 pandemic, but will require further investigation.

The current coronavirus pandemic profoundly impacts patient care, medical education, and scientific research activities. Adaptation, flexibility, creativity, and problem-solving skills are highly required and more necessary than ever before. At the same time, institutional, national, and international guidelines must be followed accurately in order to prevent further transmission of the virus. Like many ongoing studies, our randomized, double-blind, sham-controlled SCA3-tDCS trial—in which we examine the effects of cerebellar transcranial direct current stimulation (tDCS) in twenty patients with spinocerebellar ataxia type 3 (SCA3)—is affected by the health security measures dictated by the authorities and hospital board [1]. We here aim to share our experience by sketching the different scenarios that we considered for our study and end with a possible solution that may inspire investigators in ongoing ataxia trials to continue their research activities.

When the COVID-19 crisis struck the Netherlands, our study had fortunately reached its final stage. All participants had completed the 10-day regimen of daily tDCS sessions and follow-up visit after two weeks to evaluate whether modulation of cerebellar excitability is able to reduce ataxia severity and a number of non-motor symptoms. Similar to most ataxia trials, absolute change in Scale for the Assessment and Rating of Ataxia (SARA) score from baseline was chosen as the primary outcome measure [2]. Furthermore, all patients had attended long-term follow-up visits after three and six months, and sixteen of them also completed the final visit after twelve months in order to determine the precise duration of effects (if any). The remaining four subjects were scheduled in the last two weeks of March, but stringent measures prohibited these last follow-up visits to take place. As dictated by the policy guidelines of the Radboud University Medical Center, the only two exceptions for not suspending research visits included medical urgency or when their cancellation would seriously jeopardize the primary study outcome and/or study progress. We consulted the Biostatistics department of our hospital and discussed different scenarios: (1) definite cancellation of the final follow-up visits of these four patients and thus premature closure of the study, (2) postponement of these visits for a few months hoping that the crisis would soon be over (although the addition of a follow-up visit after 15, 16, or 17 months instead of 12 months might introduce heterogeneity), (3) performing an interim analysis on the short-term data and decide if live visits would be necessary, (4) completion of live assessments, with the additional risks included, if the participants are willing to come and both investigators and patients do not display COVID-19 symptoms, and (5) trying to obtain the primary endpoint via remote recordings (although we had serious concerns whether this latter option would be possible).

The statisticians ran a simulation study incorporating different scenarios and trajectories with four missing data points at twelve months follow-up but without actually using the real data. The results showed relatively limited influence of missing data on the estimated treatment effect, even in more extreme scenarios in which missing would primarily occur in one of the treatment groups. The statisticians thus argued against the completion of live assessments since the impact was deemed not large enough to justify the additional burden and risk.

Inspired by other telemedicine initiatives at our outpatient clinic and elsewhere [3], we explored the possibility of SARA home recordings by family members as a last resort strategy. Though initially disappointed about the cancellation of their visit, participants were immediately enthusiastic and willing to cooperate fully. We established an extensive set of general and item-specific instructions for the patient, the person recording the video, and a third individual necessary for the three upper limb SARA items. Participants and family members were specifically requested not to record multiple videos per item and then select the one that showed the best performance. As ataxia patients sometimes mention a diurnal variation in symptom severity, we asked to keep the time of day for the videos equal to the previous visits. Despite our initial concerns, we were pleasantly surprised by the efforts and impressed by the quality of the videos in general. When it was not possible to reliably score an item at first due to insufficient image quality, the performance (mostly of heel-shin slide or finger chase) being too slow, or the time of the recordings (of stance) being too short, the participant and family members were provided with specific instructions once more to improve and meet the quality standards. A video of the particular item recorded during one of their previous visits was sometimes also sent as a comparison. This approach allowed us to fully score the SARA and thereby obtain our primary outcome measure at the predefined point in time in all participants. We consider this experience a great example of the flexibility, creativity, and motivation of study participants, which illustrates that they can actively contribute to solutions to unforeseen problems arising during a research project.

Extraordinary times call for creative solutions that nonetheless could have important implications, not only for ongoing studies but also for future investigations. If we are able to perform interventional trials with reliable remote SARA assessments to evaluate changes in ataxia severity, this will probably benefit recruitment and retention and make studies more accessible to disabled patients. We are aware of ongoing developments in this area, such as app-based collection of home videos of patients performing a selected set of SARA items (SARA^home^), remote recordings of a SARA variant (iSARA) and of real-life gait through body-worn sensors, and Kinect-based movement tracking of SARA-inspired items in children at home (SaraHome) [4]. We are now confronted with the urgency of such tools, but until these are ready to be widely implemented, solutions such as presented here by us can be adopted instantaneously.

## References

[1] Maas RPPWM, Toni I, Doorduin J, Klockgether T, Schutter DJLG, van de Warrenburg BPC (2019). Cerebellar transcranial direct current stimulation in spinocerebellar ataxia type 3 (SCA3-tDCS): rationale and protocol of a randomized, double-blind, sham-controlled study. BMC Neurol.

[2] Schmitz-Hubsch T, Tezenas du Montcel S, Baliko L, Berciano J, Boesch S, Depondt C, Giunti P, Globas C, Infante J, Kang JS, Kremer B, Mariotti C, Melegh B, Pandolfo M, Rakowicz M, Ribai P, Rola R, Schols L, Szymanski S, van de Warrenburg BP, Durr A, Klockgether T, Fancellu R (2006). Scale for the assessment and rating of ataxia: development of a new clinical scale. Neurology.

[3] Papa SM, Brundin P, Fung VSC, Kang UJ, Burn DJ, Colosimo C, Chiang HL, Alcalay RN, Trenkwalder C, Committee MD-SI (2020). Impact of the COVID-19 pandemic on Parkinson’s disease and movement disorders. Mov Disord.

[4] Summa S, Schirinzi T, Bernava GM, Romano A, Favetta M, Valente EM, Bertini E, Castelli E, Petrarca M, Pioggia G, Vasco G (2020). Development of SaraHome: a novel, well-accepted, technology-based assessment tool for patients with ataxia. Comput Methods Programs Biomed.

